# Social Robots for Supporting Post-traumatic Stress Disorder Diagnosis and Treatment

**DOI:** 10.3389/fpsyt.2021.752874

**Published:** 2022-02-04

**Authors:** Guy Laban, Ziv Ben-Zion, Emily S. Cross

**Affiliations:** ^1^Institute of Neuroscience and Psychology, University of Glasgow, Glasgow, United Kingdom; ^2^Tel-Aviv Sourasky Medical Center, Sagol Brain Institute Tel-Aviv, Wohl Institute for Advanced Imaging, Tel-Aviv, Israel; ^3^Sagol School of Neuroscience, Tel-Aviv University, Tel-Aviv, Israel; ^4^Departments of Comparative Medicine and Psychiatry, Yale School of Medicine, Yale University, New Haven, CT, United States; ^5^The Clinical Neurosciences Division, VA Connecticut Healthcare System, United States Department of Veterans Affairs, National Center for Posttraumatic Stress Disorder, West Haven, CT, United States; ^6^Department of Cognitive Science, Macquarie University, Sydney, NSW, Australia

**Keywords:** post-traumatic stress disorder, social robots, trauma, mental health, human-robot interaction, affective computing, affective science, emotion

## Abstract

Post-Traumatic Stress Disorder (PTSD) is a severe psychiatric disorder with profound public health impact due to its high prevalence, chronic nature, accompanying functional impairment, and frequently occurring comorbidities. Early PTSD symptoms, often observed shortly after trauma exposure, abate with time in the majority of those who initially express them, yet leave a significant minority with chronic PTSD. While the past several decades of PTSD research have produced substantial knowledge regarding the mechanisms and consequences of this debilitating disorder, the diagnosis of and available treatments for PTSD still face significant challenges. Here, we discuss how novel therapeutic interventions involving social robots can potentially offer meaningful opportunities for overcoming some of the present challenges. As the application of social robotics-based interventions in the treatment of mental disorders is only in its infancy, it is vital that careful, well-controlled research is conducted to evaluate their efficacy, safety, and ethics. Nevertheless, we are hopeful that robotics-based solutions could advance the quality, availability, specificity and scalability of care for PTSD.

## 1. Introduction

Stress occurs when our dynamic biological and/or psychological equilibrium is threatened or perceived to be threatened (1, 2). The feeling of stress is prevalent and ubiquitous in our everyday lives, significantly impacting the maintenance of both physical and mental health (3), with increasing social and economic costs (4). Critically, even a single stressful event, if perceived as life-threatening (i.e., traumatic), can lead to longstanding psychopathology as exemplified by Post-Traumatic Stress Disorder (PTSD) (5). PTSD is a prevalent and severe psychiatric disorder with profound public health impact due to its chronic nature, accompanying functional impairment, and highly common comorbidities (6, 7). Existing therapeutics for PTSD show limited efficacy, presumably because they do not meet minimal quality criteria or because they attempt to treat rather than prevent the disorder (8). Furthermore, many PTSD treatments were developed without directly targeting the specific underlying mechanisms (2, 9). As both PTSD diagnosis and treatment still face significant challenges, here we aim to highlight how a novel technological solution, namely, social robots, might be able to offer assistance in the diagnosis and treatment of PTSD.

Digitization in psychiatry is gaining momentum, providing those who suffer from low mental health with an increasing array of self-help solutions, many of which are available on users' mobile devices (see 10–12). PTSD diagnosis and treatment can take many different forms, ranging from traditional questionnaires (see 13, 14) to ecological momentary assessment (EMA) (e.g., 15, 16) and intervention (EMI) (e.g., 16–18), mobile applications, virtual agents (e.g., 19–22), and exposure treatments using virtual reality (VR) devices (e.g., 23, 24). However, in the following, we argue that social robots offer another promising approach for supporting PTSD diagnosis and treatment, due to their availability, autonomy, and embodiment. Hence, this perspective paper specifically focuses on the potential application of social robots for PTSD diagnosis and treatment.

**Figure 1:**
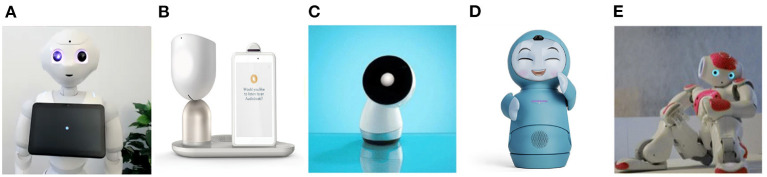
Examples of several social robotics platforms that are heavily used in research and/or have enjoyed commercial success, and are discussed in this perspective paper. **(A)** Pepper, a humanoid by SoftBank Robotics. **(B)** ElliQ, a household robot by Intuition Robotics. **(C)** Jibo, a personal home assistant robot by NTT Disruption. **(D)** Moxi, an animated household robot by Embodied. **(E)** Nao, a humanoid robot and Pepper's little sibling by SoftBank Robotics.

The definition of a social robot in this work is an autonomous machine that interacts and communicates with humans or other agents by following social behaviors and rules relevant to their role (25). Furthermore, for the purposes of this paper, we further include in our social robots definition that these machines are autonomous, and function in physical and social spaces alongside humans (26) (see Figure 1 for examples). In this paper, we wish to particularly emphasize the relevance and value of social robots' physical embodiment, as we believe this offers additional benefits beyond other digital and AI-fuelled innovations that are screen or voice-based. Social robots are attracting increasing attention for their potential use in autonomous health interventions (26). Such robots are already being applied in psychosocial interventions (27, 28), in mental health settings (29), and deployed as supportive agents to aid in rehabilitation (30, 31). Due to many social robots features, including human-like design (32–35), autonomous abilities, and high mobility (26), we suggest that some of these machines could also be well-suited to helping overcome some of the challenges of PTSD diagnosis and treatment. In this paper, we propose a novel approach for the integration of social robots in PTSD clinical management, in order to improve diagnosis and early interventions aiming to prevent and/or treat post-traumatic psychopathology. In the following, we introduce some major challenges faced in PTSD diagnosis and treatment, present an overview of recent developments in social robotics research, and reflect on the potential of these robotics developments to overcome PTSD diagnosis and treatment challenges.

## 2. Challenges for PTSD Diagnosis and Treatment

Challenges for PTSD diagnosis and treatment include the short critical time-frame for intervention after trauma exposure (36, 37); the high dependency on available qualified medical teams and trauma specialists in emergency departments (38–40); and the limited efficacy and high dropout rates of current first line pharmacological and psychological treatments (41). Moreover, when patients overcome the physical consequences resulting from the traumatic event, they often end or limit their relationship with the medical team, resulting in diminished compliance with or cessation of mental health recommendations (42).

PTSD is the only mental disorder to have a salient onset, thus providing an excellent target for secondary prevention and the mapping of pathogenic processes (43). Symptom trajectories provide an observable dimension of PTSD development or remission. Prospective studies of PTSD resulting from single traumatic incidents consistently show a progressive reduction in the symptoms' prevalence and severity during the year following trauma exposure (44–46). These early observed symptoms, seen shortly after trauma, subside in most of those initially expressing them and persist in about 30 percent of those diagnosed with PTSD 1 month after trauma (47, 48). Given the importance of interventions in the early aftermath of traumatic events, we suggest that early introduction of technology-assisted and self-help interventions should be further explored to prevent and treat post-traumatic psychopathology. Technology-assisted clinical interventions are becoming increasingly common in the health care field and in combating mental health as these interventions are often aimed at improving access to and cost-effectiveness of care (49–51).

## 3. Supporting early diagnosis in emergency departments

In emergency departments (ED) of general hospitals, diagnosing acute PTSD symptoms in trauma survivors can be a long complicated process, highly dependent on qualified human resources (e.g. trauma teams)(38–40). Moreover, the ED medical staff often focus mainly on physical injuries, prioritized by the degree of severity, disregarding mental health symptoms such as acute stress symptoms after trauma exposure. This results in patients not being diagnosed for Acute Stress Disorder (ASD) symptoms early after trauma, and receiving no intervention or treatment. If these acute stress symptoms persist for over a month after trauma exposure, the individuals are given the diagnosis of PTSD (14). Hence, it is highly important to assess early clinical symptoms shortly after trauma, and to follow-up on these throughout this early critical time-frame (36, 37). Furthermore, PTSD diagnosis rarely relies on formal and objective biological indicators, and instead indicated merely by subjective symptoms reported in clinician-administered interviews (14, 52). The clinician-administered PTSD Scale for DSM-5 (CAPS-5) is the current gold standard of PTSD assessment (14). It was designed to be administered by clinicians or clinical researchers who have a working knowledge of PTSD, but can also be administered by appropriately trained para-professionals. When necessary, to make a provisional PTSD diagnosis, the PTSD Checklist for DSM-5 (PCL-5), a 20-item self-report measure that assesses the 20 symptoms of PTSD defined by the DSM-5, can also be used (14).

While PTSD diagnosis is highly dependent on a clinician interpretation and expertise (38), it could nonetheless benefit from automation in the administration phase. For example, surveys of trauma survivors using self-reported measures (e.g., PCL-5 (13)) could be administer by a social robot, supporting human trauma teams in EDs after large-scale traumatic events, moving between trauma survivors and collecting relevant health data for diagnosing PTSD in an early stage. The current diagnosis procedure imposes a high data registration workload on medical staff, including nurses and clinicians who often demonstrate high rates of burnout due to the intense nature of EDs (53). This has serious implications, especially since trauma medical teams are often called on beyond normal working hours (54), and in case of large-scale traumatic events (e.g., large scale industrial accidents, natural disasters, terror attacks) with many trauma survivors (39). Medical teams in EDs could use the support of automatic systems to diagnose trauma survivors' mental condition in a fast and efficient way. This would provide them with time and energy to focus on other emergency medical procedures and will reduce their already-heavy workload. Trauma teams are a fundamental component for improving trauma-related care (40), hence, reducing the workload where possible can support trauma teams' performance in emergency situations. These common challenges faced in EDs and on site at traumatic events world-over also limit PTSD diagnosis, with only 7% of trauma centers reporting to be screening for PTSD (55).

Measures like the PCL-20 are constructed as self-reporting instruments that can be filled individually by trauma survivors. However, automation of some aspects of early trauma screening should also ensure that trauma survivors start the diagnosis procedure for PTSD during their hospitalization and in an early stage shortly after being exposed to a traumatic event, which should in turn reduce the patient burden of completing self-administered questionnaires in this critical time frame. As the PCL-5 is a straight-forward self-report tool that is easy to administer (13), a social robot should be able to communicate the items to most trauma survivors, and calculate a score on the spot. Moreover, due to the social robot's communication abilities, human-like design, and physical presence (see 26, 56), we propose that these self-reported items could be communicated in a more natural way. Rather than administering a questionnaire, a social robot could converse with a patient and elicit the necessary information naturally following the instrument's items (e.g., 34, 57). Using the robot's interactivity features, the social robot can update the system and prioritize fast and personalized reactions from relevant medical professionals (e.g., a psychiatrist, social worker, psychologist) for further elaborate diagnosis and early intervention.

Previous studies and ecologically valid study reports positive evidence for the use of social robots in autonomous health data acquisition among hospitalized patients. Moreover, these studies reported for social robots administrating health data acquisition in a variety of settings such as hospitals, homes, schools, and nursing homes (58–60). A randomized controlled cross-over trial with a social robot (Pepper, SoftBank Robotics, see Figure 1) and a nurse administering three questionnaires (52 questions in total) showed minimal differences in health data acquisition effectiveness between the two conditions (the Pepper robot vs. the nurse). Moreover, the study results demonstrated that the social robot was accepted by the patients (older adults) (58). The study suggests that social robots may effectively collect health data autonomously in public settings, and assist medical teams in diagnosing patients. As mentioned earlier, using a social robot for surveying trauma survivors via a self-reported tool can reduce the load on the trauma medical teams and ensure that relevant data for the diagnosis will be collected and reported in the relevant medical system on time for early intervention.

It is important to highlight that while there is vast evidence in the social robotics and human—robot interaction research literature for the effect of social robots on human's behavior in health settings (e.g., 28, 29, 61), and on self-disclosure in particular (34, 62–66), eliciting information from trauma survivors (especially regarding the trauma and the associated affect) is substantially different and will impose different and new challenges. This will require further investigation via future empirical research, as it is vital to understand disclosure to a social robot (and how different it is from disclosure to a human or disembodied technologies) when people are in a hyper-vulnerable state.

## 4. Overcoming logistical barriers

Following the administration of acute medical treatment, immediately following a trauma, several further logistical barriers exist that can prevent trauma survivors from receiving proper mental health diagnosis and intervention. These can be personal (e.g., living in rural areas with limited local mental health providers, limited mobility, language barriers, legal status, poor relationships and communication with providers, fragmentation of care) or professional (e.g., lack of support from the employer, lack of time, high responsibility, isolated employment) (67). These logistical barriers can be crucial considering the shortage of mental health professionals, especially in rural and difficult-to-access regions (4, 68, 69). An example is the barriers experienced by active-duty and ex-serving military personnel who suffer from PTSD. Studies involving active-duty infantry US soldiers demonstrate that 28% of soldiers met self-reported criteria for PTSD or major depressive disorder in the post-deployment period (70). Nevertheless, less than 40% of soldiers with mental health problems utilize mental health services, and only 50% seek intervention following a clinical referral (70). Active-duty soldiers report for logistical barriers when seeking mental health. These include difficulties in arranging appointments, lack of mental health professionals and/or limited availability in remote military bases, and lack of opportunities to see mental health professionals in their limited time outside military basis (71, 72). Importantly, active-duty soldiers are not the only people who suffer from limited access to mental health services. People who live in rural areas, or in regions with limited mobility, also frequently report having limited access to mental health services (4, 67, 73).

While a social robot can not and would not replace a human clinician in these settings, it could possibly be situated in these unique hard-to-reach environments, aiming to expand some aspects of mental health service delivery. These aspects include, but not limited to, local clinics in rural areas, far military bases, community centers, and homes of people with limited mobility. A social robot could collect health-related data in one's home or another familiar environment, monitor and report symptoms, and potentially offer early intervention in familiar settings. Deploying social robots in such a way could provide cost-effective mental health support, offering solutions to those with limited opportunities to access mental health services in their everyday settings. Accordingly, clinical symptoms of trauma survivors could be monitored early after trauma exposure (6, 74), and clinical teams could prioritize those who are at high-risk for PTSD development. This in turn would allow employment of early interventions aiming to prevent the development of the chronic disorder, which is more efficient than trying to treat chronic PTSD (8). Furthermore, small accessible social robotic devices designed for the home—such as ElliQ (Intuition Robotics), Moxi (Embodied), and Jibo (NTT Disruption) (see Figure 1)—could be placed in people's homes to monitor symptoms of trauma survivors with limited mobility. These social robots are easy to operate, easy to transport, and can elicit meaningful responses from humans in relevant settings (26, 75, 76).

A social robot in these settings can also be remotely operated by a clinician from afar, serving as a telepresence medium to provide access to professional mental health care providers in isolated settings (see 77, 78). For example, SoftBank Robotics recently introduced a new telepresence feature for their Pepper robot (79, 80). In contrast to other telepresence robots that are merely an extension of the telepresenced human, here the human telepresenced through the social robot shares a body with another social entity - such as Pepper, the humanoid social robot. This feature could introduce valuable opportunities for using social robots for PTSD, where they can perform both autonomously and/or be controlled by proxy. Therefore, aided by their physical embodiment, social robots offer the potential to provide human-mediated care by proxy as well as by using their autonomous programming to administer clinical management tasks independently when needed.

## 5. Overcoming Social Barriers

Extending from logistical barriers, social robots can also help to overcome social barriers for those seeking mental health treatment for PTSD. Some trauma survivors consider their hospitalization to be traumatic, hence they tend to avoid visiting or consulting with clinicians (81). Others avoid seeking mental health treatment at all due to personal internal social barriers such as stigma, isolation, stress, prejudice and feelings of shame (67, 82) associated with traumatic experiences (70, 82–86). Indeed, evidence of active-duty members and veterans demonstrating an unwillingness to discuss their mental health or emotions with medical teams due to prejudice and stigma has been well-documented (70, 82). Individuals with combat-related PTSD often feel strong negative emotions (e.g., anger, guilt, shame) in relation to the trauma and their subsequent mental condition. For examples, feeling of shame were associated with worse clinical outcomes in veterans with PTSD, specifically increase in suicidal ideation (85). Furthermore, sexual assault victims exhibited difficulties to discuss their traumatic events in both formal and informal settings (87). Finally, other individuals are not willing to receive mental health assistance mainly due to lack of support from family, friends, and their community (67).

We suggest that a social robot can potentially bypass some of the above-mentioned social barriers, and encourage individuals to report and treat their post-traumatic symptoms. We see compelling evidence for people being willing to disclose sensitive information, including stressors and mental health symptoms, to avatars and virtual agents. For example, a study by Utami et al. (19) explored the reactions of older adults when having “end-of-life” conversations with a virtual agent. The study's results show that all study participants were comfortable discussing with the agent about death anxiety, last will and testament, providing compelling evidence for the potential utility of artificial agents in these complex socioemotional domains. Another study by Lucas et al. (20) employed a virtual agent that affords anonymity while building rapport to interview active-duty service members about their mental health symptoms after they returned from a year-long deployment in Afghanistan. The study reports that participants disclosed more symptoms to a virtual agent interviewer than on the official Post-Deployment Health Assessment (PDHA), and than on an anonymized PDHA. Moreover, the results of a larger sample experiment with active-duty and former service members reported a similar effect (20).

Furthermore, another recent study (34) examined the extent to which social robot and disembodied conversational agent (voice assistant) can elicit rich disclosures, and accordingly might be used to support people's psychological health through conversation. The study reported that a social robot (NAO, SoftBank Robotics, see Figure 1) was successful in eliciting rich disclosures from human users, evidenced in the information that was shared, people's vocal output, and their perceptions of the interaction (34). This is in line with additional works that report different behavioral and emotional effects when communicating with social robots, and increased willingness of participants to disclose information and emotions in the presence of embodied artificial agents (e.g., 63, 65, 88–91). While participants were aware of many of the obvious differences between speaking to a humanoid social robot (NAO, SoftBank Robotics) compared to a disembodied conversational agent (Google Nest Mini voice assistant), their verbal disclosures to both were similar in length and duration (34). Another study (92) demonstrated positive responses of human users to a humanoid robot taking the role of couples counselor, aiming to promote positive communication. It is of note that the robot also played a meaningful role in mediating positive responses (in terms of behavior and affect) within the couples' dyadic interaction in this same study. While social robots obviously can not offer the same opportunities for social interaction and engagement as humans (33), their cognitive architectures and embodied cognition can nonetheless elicit socially meaningful behaviors from humans (93). As such, they can afford valuable opportunities for engagement with humans when introduced in specific contexts, and in careful ethically responsible ways (94).

## 6. Discussion and Conclusions

Through this paper, we aimed to introduce several challenges related to PTSD diagnosis and treatment, and highlight suitable opportunities to address them by introducing social robots in PTSD diagnosis and treatment. As it is crucial to diagnose acute PTSD early after trauma, social robots can support clinical assessments of trauma survivors during the hospitalization phase. They may also aid trauma teams in EDs by reducing some of their stress and burden during busy times (39, 40). As social robots can support high fidelity data collection and on-line, on-going analysis of human behavior, emotions, and physiological reactions, they might have the potential to support early diagnosis of PTSD among trauma survivors. Finally, social robots can assist with overcoming several logistical and social barriers that trauma survivors face when required to monitor symptoms and when seeking mental health interventions (67, 73, 81, 82).

We clearly acknowledge that various screen-based (or virtual), non-embodied technologies can also assist with some of the challenges, for example, via the use of EMA and EMI methodologies (e.g., 15, 17), or through use of a virtual agent on one's mobile device (e.g., 19, 20). While these kinds of tools and instruments might be useful and widely available, social robots provide an additional benefit through their embodiment, in that they have the potential to communicate and interact with people in a more socially meaningful way by initiating interactions more naturally than mobile devices, and providing rapid and responsive ecological momentary interventions in users' natural physical settings. While mobile apps require the user to take a certain initiative to log information, take action, or respond to a notification, social robots can elicit interactions more naturally due to their design, animated behavior, and social roles (see 26, 95, 96). This would be extremely helpful when monitoring symptoms for trauma survivors since they often prefer to refrain from discussing the trauma (67, 82). In fact, most EMA and EMI mobile solutions for self-monitoring are highly dependent on users' initiative and responsibility (see 97–99), which can be very challenging after experiencing a traumatic event. Moreover, EMA's and EMI's repetitive nature could be further triggering when addressing aspects related to traumatic events (see 98). Accordingly, social robots might just fall at the ideal intersection between being an autonomous and physically present technology that can capture emotion and information while also being able to demonstrate social and cognitive cues that might help to elicit rich and valuable disclosures from these patients. We do not argue that social robots are a perfect solution, but rather that they could potentially help overcome some of the barriers that other solutions might still struggle with.

It should also be mentioned that while social robots are indeed more expensive and less readily available than smartphone devices, they remain reasonably employable in social spaces (see 100), and can take on a cost-effective and user-friendly embodiment of a household robot (see 26, 75). To sum up, while smartphone applications have clear benefits in terms of availability, cost and scalability, in our perspective social robots' physical embodiment and cognitive architectures could support richer interactions with human users, which in turn could potentially help to overcome some of the logistical and emotional barriers of PTSD diagnosis and treatment.

To the best of our knowledge, no social robotics studies to date have been conducted with trauma survivors or individuals diagnosed with PTSD. Nonetheless, a study by Nomura and colleagues (101) provides evidence for the benefits of employing social robots for minimizing social tensions and anxieties. Through this work, the authors showed that participants with higher social anxiety tended to feel less anxious and demonstrated lower tensions when knowing that they would interact with a robot, as opposed to a human, in a service interaction. In addition, the authors suggested that an interaction with a robot elicited lower tensions compared to an interaction with another person, regardless of one's level of social anxiety. Extrapolating to PTSD settings, it is reasonable to assume that social robots might support trauma survivors with overcoming their social barriers to disclose and monitor their symptoms over time. Whether these robotic agents are situated at home, the local clinic, community center or hospital, they hold the potential to reach patients and invoke authentic reactions that could be critical for early diagnosis and treatment of PTSD.

Due to the lack of empirical work on this issue, in this perspective paper, our primary aim was to address the potential for introducing social robots for PTSD diagnosis and treatment, based on evidence gathered from a variety of different applications and perspectives from both clinical and non-clinical contexts. As such, we would like to stress that the ideas presented in this perspective paper are at a very early stage, based on studies with a variety of populations, and will need to be carefully and ethically tested before applying them to interventions with people in a hyper vulnerable state (such as those who experienced traumatic events or have already been diagnosed with PTSD). Studies looking into this should start by testing participants after experiencing trauma but without demonstrating PTSD symptoms or with subthreshold symptoms. When seeing good and valid results, studies could then carefully and responsibly move on to being replicated with clinical populations carefully. These sorts of trials should be accompanied/supervised or monitored by a mental health professional to ensure that participants do not experience negative effects.

Most of the preventive therapies for PTSD to date have been developed without directly documented neurocognitive targets (9). The currently most effective treatment for PTSD (cognitive behavioral training (CBT)) (102) was conceived entirely on psychological grounds. Similarly, trials of the most effective drugs for PTSD, selective serotonin reuptake inhibitors (SSRIs) (103), were based on these drugs' observed antidepressant effect; the recognition that the serotoninergic system is involved in the biology of PTSD only came afterward. Despite the abundance of biological insights into PTSD that have been achieved, the development of treatments for PTSD has not been different from the history of much of medicine: effective agents are discovered by serendipity, and their biological mechanisms of action are only clarified later. As social robots could potentially hold out the prospect of providing a nuanced and novel solution to some of the challenges that PTSD diagnosis and treatment are facing, future research should be appropriately conducted to test this premise.

## Data Availability Statement

The original contributions presented in the study are included in the article/supplementary material, further inquiries can be directed to the corresponding author.

## Author Contributions

GL and ZB-Z conceptualized the paper and wrote the first draft. EC supervised and revised. All authors edited and approved the final version.

## Funding

The authors gratefully acknowledge funding from the European Union's Horizon 2020 Research and Innovation Programme under the Marie Sklodowska-Curie to ENTWINE, the European Training Network on Informal Care (Grant agreement no. 814072), the European Research Council (ERC) under the European Union's Horizon 2020 Research and Innovation Programme (Grant agreement no. 677270 to EC), and the Leverhulme Trust (PLP-2018-152 to EC).

## Conflict of Interest

The authors declare that the research was conducted in the absence of any commercial or financial relationships that could be construed as a potential conflict of interest.

## Publisher's Note

All claims expressed in this article are solely those of the authors and do not necessarily represent those of their affiliated organizations, or those of the publisher, the editors and the reviewers. Any product that may be evaluated in this article, or claim that may be made by its manufacturer, is not guaranteed or endorsed by the publisher.
